# Insights into the clinical value of cyclin-dependent kinase 5 in glioma: a retrospective study

**DOI:** 10.1186/s12957-015-0629-z

**Published:** 2015-07-25

**Authors:** Ruan Yushan, Chen Wenjie, Huang Suning, Dang Yiwu, Zhong Tengfei, Wickramaarachchi Mihiranganee Madushi, Luo Feifei, Zhang Changwen, Wen Xin, Gopaul Roodrajeetsing, Li Zuyun, Chen Gang

**Affiliations:** Department of Neurosurgery, First Affiliated Hospital of Guangxi Medical University, 6 Shuangyong Road, Nanning, Guangxi Zhuang Autonomous Region 530021 People’s Republic China; Department of Pathology, First Affiliated Hospital of Guangxi Medical University, 6 Shuangyong Road, Nanning, Guangxi Zhuang Autonomous Region 530021 People’s Republic China; Department of Radiology, First Affiliated Hospital of Guangxi Medical University, 6 Shuangyong Road, Nanning, Guangxi Zhuang Autonomous Region 530021 People’s Republic China

**Keywords:** CDK5, Glioma, Immunohistochemistry, Clinical features

## Abstract

**Background:**

Previous studies suggested that expression of cyclin-dependent kinase 5 (CDK5) may promote the migration and invasion of human glioma cells. In this study, we aimed to evaluate the clinical value of CDK5 in different grades of glioma in relation to Ki-67 labeling index (LI).

**Methods:**

We firstly assessed by immunohistochemistry the expression of CDK5 in 152 glioma tissues and 16 normal brain tissues and further explored the relationship between CDK5 expression and other clinical features.

**Results:**

The positive ratio of CDK5 in gliomas (57.2 %) was higher than that in normal brain tissues (12.5 %, *P* = 0.001). Difference of CDK5 expression among four World Health Organization (WHO) grades was statistically significant (*P* = 0.001). The significant differences of CDK5 expression were also observed between WHO I glioma (34.8 %) and WHO III glioma (62.5 %), as well as WHO IV glioma (82.8 %; *P* = 0.026, *P* < 0.001, respectively). Furthermore, Spearman’s rank correlation confirmed that CDK5 was positively correlated with the pathological grade of glioma (*r* = 0.831, *P* < 0.001). The CDK5 expression was also positively correlated with Ki-67 LI (*r* = 0.347, *P* < 0.001).

**Conclusions:**

The current result suggests that CDK5 may play an essential role in the tumorigenesis and aggressiveness of gliomas.

## Background

Gliomas, the most frequent intracranial malignant tumor, are categorized as grade I to grade IV based on histological and clinicopathological criteria, as provided by the World Health Organization (WHO) [1]. Gliomas of grade I are often considered to be benign. They are commonly curable with thorough surgical resection and seldom have the possibility of evolving into higher-grade lesions. However, gliomas of grade II or III demonstrate the invasion and progression to higher-grade lesions with a poor consequence. WHO grade IV gliomas, the most aggressive form, have a dismal outcome [2]. During the past decade, understanding of gliomagenesis has expanded widely. Various molecular aberrations of gliomas harbor valuable information for diagnosis, prognosis, or prediction, specifically comprising the combined loss of chromosome arms 1p and 19q, TP53 mutation, the incidence of isocitrate dehydrogenase 1 (IDH1) mutation, and the amplification of epidermal growth factor receptor (EGFR) and copy number aberrations of chromosomes 7 and 10 [3–6].

Cyclin-dependent kinase 5 (CDK5), which is a proline-directed serine/threonine kinase, expresses predominately in mature neurons and is involved in neurite extension, neuronal migration, and neuronal differentiation [7]. Cellular stress can induce the cleavage of p35 by the Ca^2+^-dependent protease calpain to p25, which aberrantly activates CDK5, thereby promoting the phosphorylation of substrates implicated in neurodegeneration [8, 9]. Actually, elevated CDK5 expression has been detected in different classes of cancers, such as lung, pancreatic, neuroendocrine thyroid, and breast cancer [10–13]. Previously, activity of CDK5 has been noted in oligodendrocytes [14]. Additionally, evidence points that CDK5-mediated phosphorylation of PIKE-A (isoform A of phosphatidylinositol 3-kinase enhancer), which is a novel pro-oncogenic and antiapoptotic element that activates protein kinase B (AKT) pathway and stimulates cell growth, promotes cell migration and invasion in glioblastomas [15]. More recently, CDK5-induced p-PPARγ has been reported to downregulate GFAP which preserves shape and arbor of astrocyte processes contributing to the cellular mechanical strength of the astrocyte [16]. These aforementioned studies support the hypothesis that expression of CDK5 promotes the migration and invasion of human glioma cells. To our knowledge, there has been no study investigating the different expression of CDK5 between normal human brain and glioma tissues in a large number of patients. Thus, the current study was to investigate the possible role of CDK5 in the tumorigenesis and aggressiveness of glioma and emphasized the relationship of CDK5 expression with Ki-67 labeling index (LI), which represents the status of tumor cell proliferation.

## Methods

### Patients

This retrospective study included a total number of 152 glioma samples that were surgically resected from the First Affiliated Hospital of the Guangxi Medical University (Nanning, Guangxi, China) during the period from January 2008 until September 2013. The patients in the current study included 106 males (69.7 %) and 46 females (30.3 %) with a mean age of 40.50 ± 16.6 (range, 7–75 years old). According to the classification criteria of nervous system tumors (WHO, 2007), 23 cases were WHO grade I as pilocytic astrocytomas, 44 cases were grade II (including 21 cases of fibrillary astrocytoma, 16 cases of protoplasmic astrocytoma, and 7 cases of oligodendroglioma), 56 cases were WHO III (including 42 cases of anaplastic astrocytoma and 14 cases of anaplastic oligodendroglioma), and 29 cases were WHO IV (all glioblastoma). Another 16 cases of normal frontotemporal brain tissues under intracranial decompression were collected from the First Affiliated Hospital of the Guangxi Medical University as a control group. Thirteen male and three female individuals were enrolled in the study as healthy controls, with ages between 23 and 59 years old (with the median age of 48 and mean age of 43.75 ± 15.34). The clinical data was obtained from the patients’ medical records and included age, gender, and WHO grade.

### Immunohistochemistry

Polyclonal antibody anti-CDK5 (sc-173, Santa Cruz Santa Cruz Biotechnology Inc., CA, USA) and monoclonal antibodies Ki-67 (Beijing Zhongshan Jinqiao Inc., Beijing, China) were used to perform the immunohistochemical detection as previously described [17, 18]. To score CDK5 as immunopositive staining, the positive cells are shown as a yellow to brown color of the nucleus and/or cytoplasm. One hundred cells from 10 representative regions from each case were evaluated. The immunohistochemical results were assessed based on the immunodetection of stain intensity and number of positive cells. The results of staining were evaluated by each author and an final agreement regarding controversial cases was reached at a multiheaded microscope. CDK5 expression was classified semiquantitatively according to the following criteria: No staining was recorded as 0; weak staining with focal or fine granular morphology was considered as 1; linear or cluster, strong staining was 2; and diffuse, intense staining was considered as 3. As for the positive cells in each case, the score ranged from 0 to 3 in percentage: 0 was when no staining was observed, 1 presented as less than 30 % cells were stained, and 2 was from 30 to 70 %. When more than 70 % cells were positive, it was recorded as 3. Finally, the samples were categorized as positive and negative based on the sum of the scores as follows: 0–2: negative, 3: weakly positive (+), 4: moderately positive (++), and 5–6: strongly positive (+++). Any score >3 was considered as positive expression in the current study. For detection of Ki-67 LI, the positive cells were shown as the distribution in the nuclei. With the formula (number of positive cells/total number of the cells × 100 %), the Ki-67 LI was calculated by observing at least 10 representative visions at high magnification (40 × 10) [18, 17, 19].

### Statistical analysis

For statistical analysis, SPSS 20 software was used. The difference of CDK5 expression between two patient subgroups was determined by using chi-square test. Kruskal-Wallis *H* test was performed to detect the differences between CDK5 expression and pathological grades of glioma. The significance of difference of LI between two groups was analyzed by unpaired Student’s *t* test. The statistical significance of the difference of LI between more than two groups was assessed by using ANOVA test. The predictive values of CDK5 and Ki-67 LI in diagnosis and tumor differentiation were evaluated by the receiver operating characteristic (ROC) curves. Spearman’s correlation was used to study the correlation between CDK5 expression, Ki-67 LI, and tumor grade. *P* values were two-sided, and significance level of *P* < 0.05 was set up in all tests.

## Results

### Immunohistochemical location of CDK5 expression

**Fig. 1:**
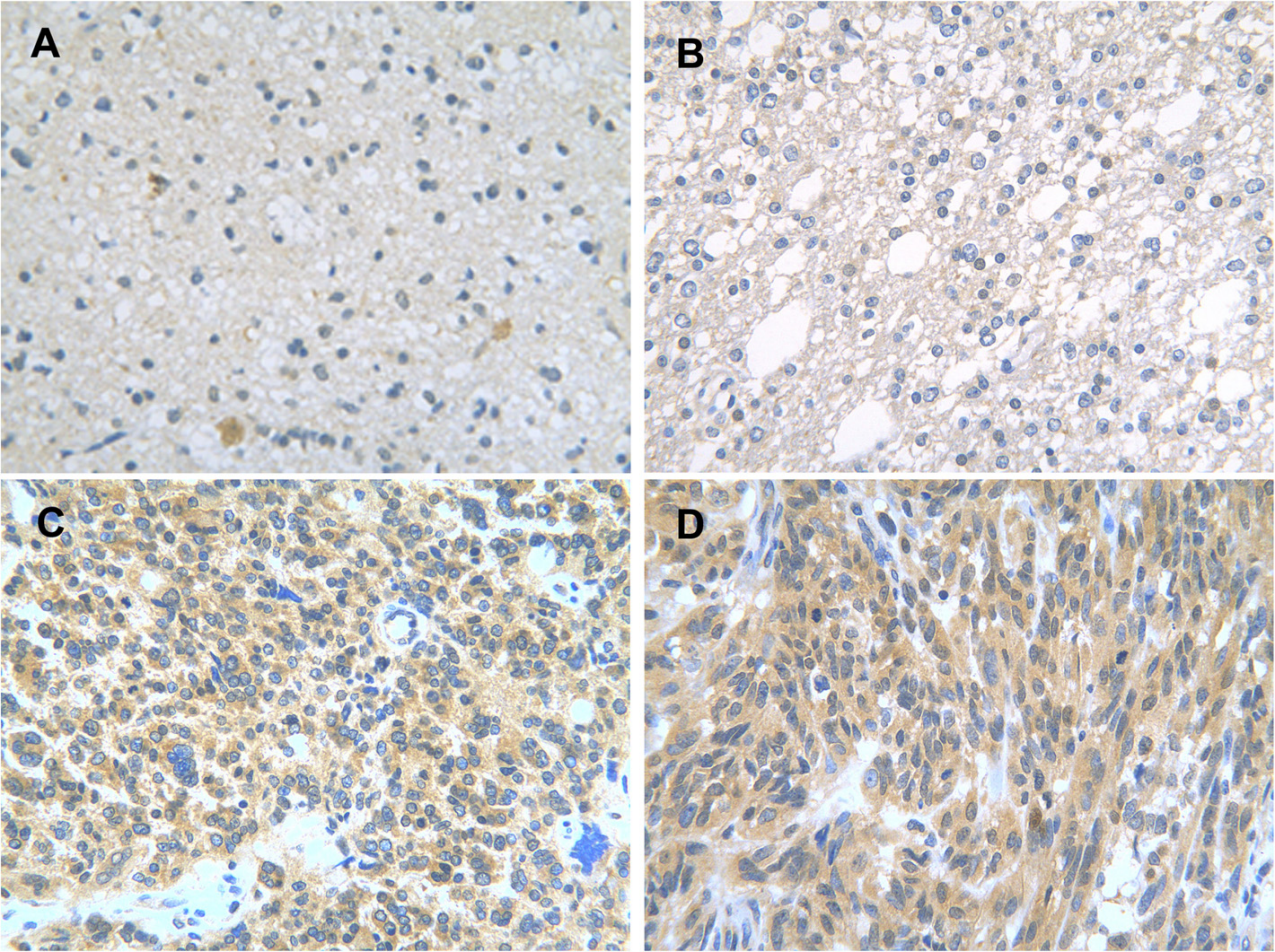
Expression of CDK5 in different grade glioma tissues. **a** WHO I glioma; **b** WHO II glioma; **c** WHO III glioma; **d** WHO IV GBM

Immunohistochemical staining results showed that CDK5 protein was expressed in glioma cell cytoplasm (Fig. 1).

### CDK5 expression in gliomas

**Fig. 2:**
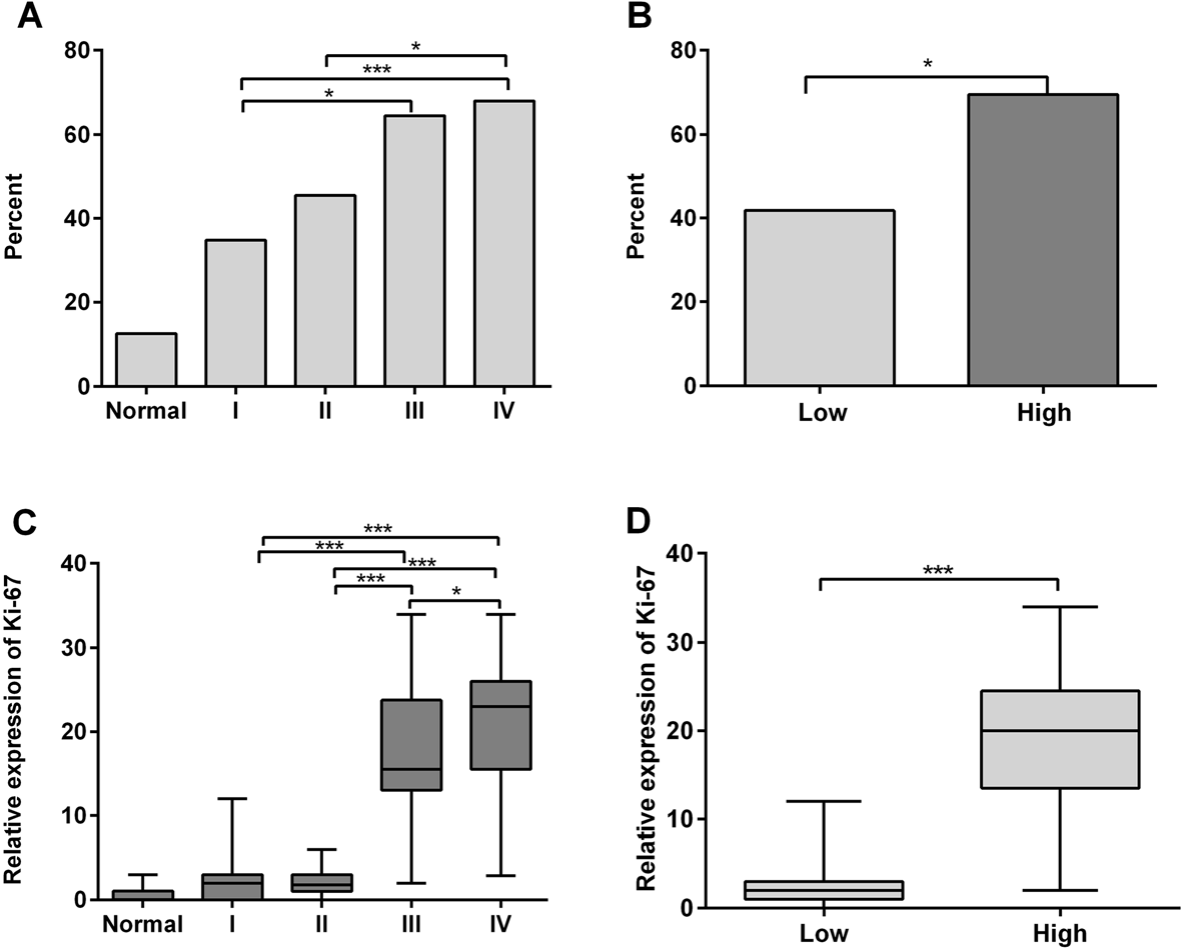
The implication of CDK5 expression and Ki-67 LI in different tissues. **a** CDK5-positive expression was higher in glioma tissues than in normal brain tissues (*P* = 0.002). The positive rate of CDK5 expression in WHO III and WHO IV was also significantly higher than in WHO I (*P* = 0.021, *P* = 0.020, respectively); **b** CDK5 expression was significantly higher in high-grade gliomas than low-grade gliomas (*P* < 0.001); **c** the significant differences of Ki-67 LI among the different groups; **d** higher Ki-67 LI was detected in high-grade gliomas than in low-grade gliomas (*P* < 0.001). *P < 0.05;**P < 0.01;***P < 0.001

CDK5-positive expression (57.2 %) was higher in glioma tissues than in normal brain tissues (12.5 %, *Z* = −3.400, *P* = 0.001). The CDK5-positive expression in WHO III glioma (62.5 %) and WHO IV glioma (82.8 %) was observed to be higher when compared to WHO I glioma (34.8 %; *P* = 0.026, *P* < 0.001, respectively, Fig. 2a). The results disclosed a tendency of CDK5-positive ratio to increase in high-grade glioma compared to low-grade glioma (*Z* = −3.406, *P* = 0.001, Fig. 2b). The differences among four grades were also statistically significant (*F* = 15.482, *P* = 0.001, Table 1) as assessed by Kruskal-Wallis *H* test. Spearman’s rank correlation confirmed that CDK5 was positively correlated with the pathological grade of glioma (*r* = 0.277, *P* = 0.001).

**Table 1:** Association of CDK5 expression and Ki-67 LI with clinicopathological features

Parameters	Total (*n*)	Expression of CDK5 *n* (%)		*Z*	*P*	Ki-67 relevant expression (2^−ΔCq^)	*t*	*P*
		Negative	Positive			Mean ± SD		
Tissue								
Normal tissue	16	14 (87.5 %)	2 (12.5 %)	−3.400	0.001	0.6062 ± 0.9518	−12.464	<0.001
Glioma	152	65 (42.8 %)	87 (57.2 %)			11.4053 ± 10.2714		
Gender								
Female	49	23 (46.9 %)	23 (53.1 %)	−0.014	0.989	10.6449 ± 10.4964	0.216	0.829
Male	119	56 (47.1 %)	63 (52.9 %)			10.2664 ± 10.2259		
Age (median)								
≤41.5	84	34 (40.5 %)	40 (59.5 %)	−1.695	0.09	11.5940 ± 10.6489	1.542	0.125
>41.5	84	45 (53.6 %)	39 (46.4 %)			9.1595 ± 9.8001		
Grading1								
I–II	67	39 (58.2 %)	28 (41.8 %)	−3.406	0.001	2.0373 ± 1.8660	−18.988	<0.001
III–IV	85	26 (30.6 %)	59 (69.4 %)			18.7864 ± 7.85763		
Grading2								
I	23	15 (65.2 %)	8 (34.8 %)	15.482^a^	0.001	2.0000 ± 2.5045	104.022^b^	<0.001
II	44	24 (54.5 %)	20 (45.5 %)			2.0568 ± 1.4609		
III	56	21 (37.5 %)	35 (62.5 %)			17.4571 ± 7.3644		
IV	29	5 (17.2 %)	24 (82.8 %)			21.3621 ± 8.2639		
CDK5								
Low	79					6.5380 ± 8.4147	−4.928	<0.001
High	89					13.7843 ± 10.6130		
Ki-67 (median)								
Low	84	54 (64.3 %)	30 (35.7 %)	−4.469	<0.0001			
High	84	25 (29.8 %)	59 (70.2 %)					

^a^Kruskal-Wallis *H* test was performed

^b^One-way analysis of variance (ANOVA) test was used

### The relationship between CDK5 and Ki-67 LI

**Fig. 3:**
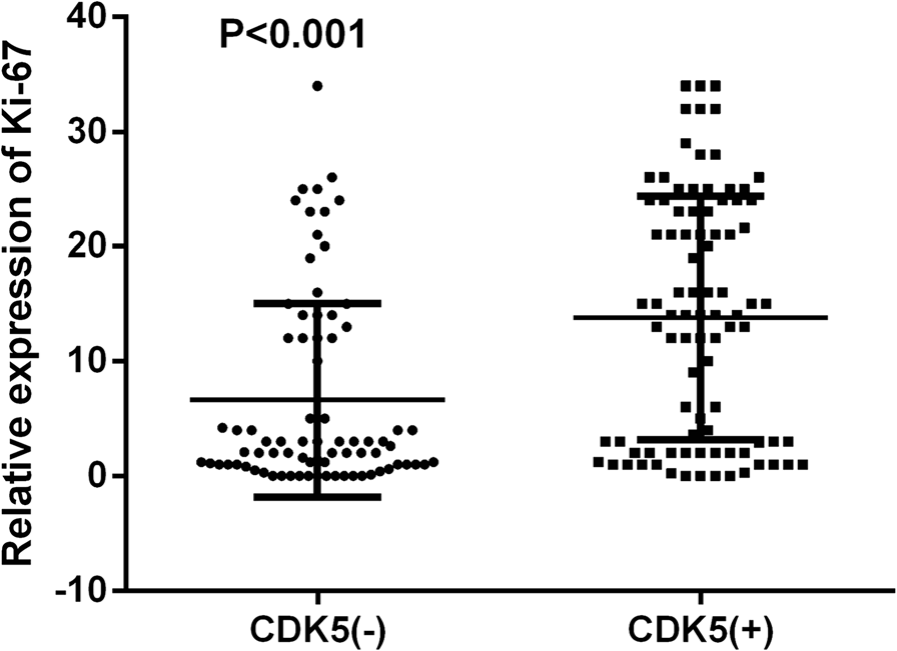
The comparison of Ki-67 LI between CDK5-positive and CDK5-negative group. The CDK5-positive group revealed higher Ki-67 compared to the negative one (*P* < 0.001)

The result of Ki-67 LI was shown in Table 1. The glioma tissues showed significantly higher Ki-67 LI than that in normal brain tissues (11.41 ± 10.27 vs 0.61 ± 0.95, *P* < 0.001). Ki-67 LI also showed significant differences among the four WHO grades (*P* < 0.001, Fig. 2c). Ki-67 LI was observed to be higher in high-grade glioma tissues than in low-grade glioma tissues (18.79 ± 7.86 and 2.03 ± 1.87, *P* < 0.001, Fig. 2d). The significant correlation between Ki-67 LI and the histological grade was found (*r* = 0.831, *P* < 0.001). The Ki-67 LI also showed a significant difference between CDK5-positive group and CDK5-negative group (13.78 ± 10.61 vs 6.54 ± 8.41; *P* < 0.001, Fig. 3). There was a significant correlation for CDK5 expression and Ki-67 LI in all tissues (*r* = 0.347, *P* < 0.001).

### The predictive value of CDK5 for the occurrence and progression in glioma

**Fig. 4:**
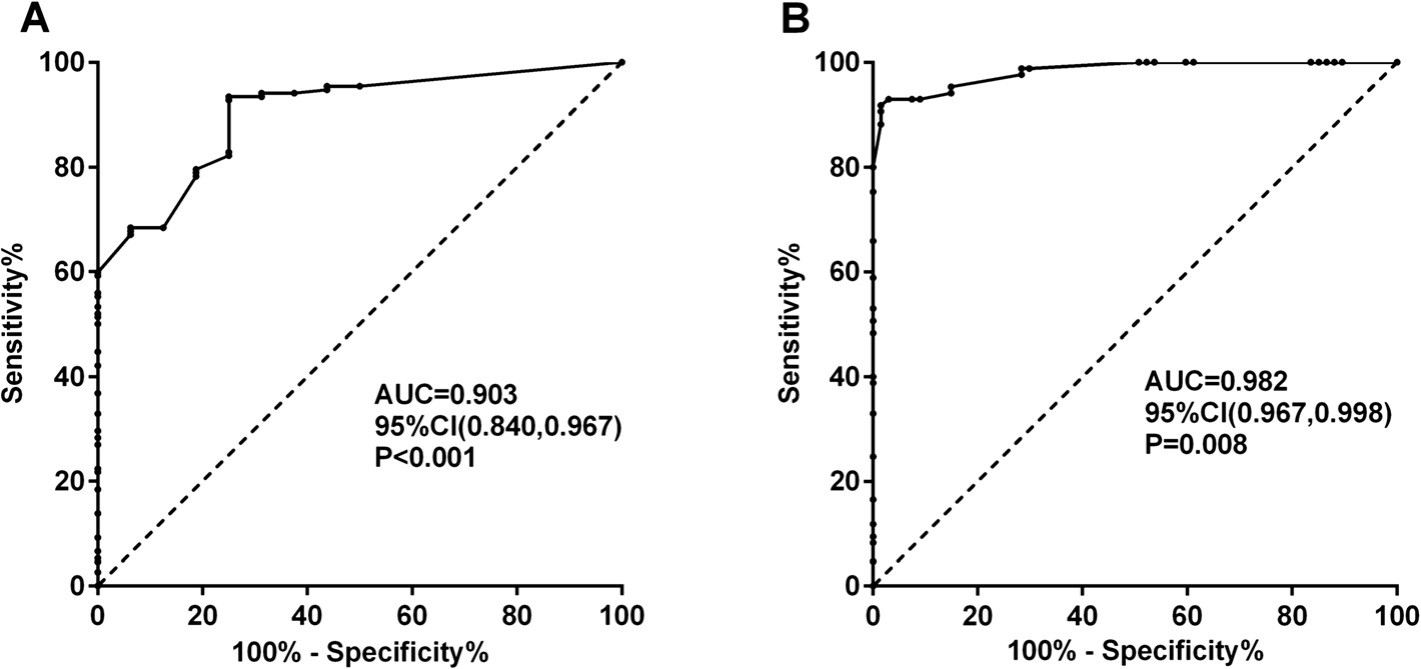
The ROC curves for the predicative value of Ki-67. **a** AUC of Ki-67 LI for diagnosing gliomas was 0.903 (95 % CI 0.840, 0.967; *P* < 0.001); **b** Ki-67 LI as ≥7.5 could predict high-grade WHO glioma (AUC = 0.982, 95 % CI 0.967, 0.998; *P* = 0.008)

To investigate whether CDK5 expression levels have the diagnostic value for glioma, ROC was performed. The most notable finding was that CDK5 could be a significant effect for diagnosing gliomas (AUC = 0.724, 95 % confidence interval (CI) 0.610, 0.837; *P* = 0.003). CDK5 expression level significantly contributed to diagnosing high-grade glioma (AUC = 0.666, 95 % CI 0.584, 0.749; *P* < 0.001). The ROC curve indicated that Ki-67 LI ≥ 0.7 could significantly diagnose gliomas with 93.42 % sensitivity and 75 % specificity and with AUC as 0.903 (95 % CI 0.840, 0.967; *P* < 0.001, Fig. 4a). Additionally, the significance also reached in the predictive effect of glioma histological grade; Ki-67 LI as ≥7.5 could predict high-grade WHO glioma (AUC = 0.982, 95 % CI 0.967, 0.998 with 91.76 % sensitivity and 98.5 % specificity, Fig. 4b).

## Discussion

Glioma encompasses different histological subtypes with high variability in prognosis and accounts for almost 80 % of primary malignant brain tumors [20]. At present, the causes for glioma tumorigenesis and aggressiveness remain unclear. Recently emerging researches have investigated the expression of novel biomarkers in glioma. For example, the hyper-methylation statuses of EGFR and methyl-guanine-DNA methyltransferase (MGMT) have been shown to play vital roles in glioma progression [21].

CDK5, one of the major kinases activated by its regulators p35 or p39, directly phosphorylates various residues and simultaneously regulates various substrates [7]. Evidence indicates that CDK5 may have extra neuronal functions that comprise transcript-selective translation control, glucose-inducible insulin secretion, vascular angiogenesis, cell adhesion, migration, and wound healing [22, 23]. Previous studies demonstrated that CDK5 overexpression was implicated in the tumorigenesis and aggressiveness in several malignancies, for instance lung, pancreatic, neuroendocrine thyroid, and breast cancer [13, 12, 11, 10]. Similar results were achieved in our current study that CDK5 expression was significantly upregulated in gliomas tissues, as compared to normal brain tissues. Further, our study found that CDK5 might be a significant diagnostic factor for glioma with a large population (*n* = 152) tested. Consistent with our study, CDK5 expression was observed to be overexpressed in NSCLC and breast cancer, which implies that CDK5 serves as an important role in the tumorigenesis of different malignancies [24, 25]. Evidence has shown that CDK5 can downregulate the tumor suppressor DLC1 and target Dab1 and p53 in cancers, acting as a pro-oncogenic factor [26–28]. Moreover, the increase of CDK5 is involved in the pro-oncogenic pathways by targeting Ral and FAK [11, 29]. CDK5 is also involved in cell cycle of cancer by inactivating pRB [12]. With further studies of CDK5, the researchers speculated the potential roles of CDK5 in glioma. Tsai et al. were the first to report that CDK5 protein and kinase activity were lacking in a human GBM cell line [30]. Later, Gao et al. identified that CDK5-T33 could suppress the change of shape, loss of adhesion, and apoptosis that characterize the response of U373 glioma cells to heat stress [31]. Researchers have also found that CDK5 protein expression is related with apoptosis in human glioblastoma multiforme [32]. In light of our study and previous reports, we hypothesized that CDK5 may act as a pro-oncogenic factor in gliomagenesis. However, additional functional studies in vitro and in vivo are needed for the understanding of the role of CDK5 in gliomagenesis.

Further, we continued to investigate the relationship between CDK5 expression and clinicopathological factors of gliomas, mainly the tumor grades. In the current study, we observed that the increasing expression of CDK5 protein was significantly related to the pathological grade in our set of 152 glioma cases. In accordance with our study, Catania et al. conducted the study that CDK5 expression in the astrocytomas of grades II–IV was consistently stronger than that in a single grade I pilocytic astrocytoma, which was the first study to examine CDK5/p35 expression in human astrocytic tumors of varying grades. Nevertheless, the study only enrolled a small size of 12 human astrocytic tumor specimens [32]. Liu et al. identified that CDK5-mediated phosphorylation of PIKE-A could activate AKT and enhance cell growth, as well as induce glioblastomas cell migration and invasion [15]. Previous studies also confirmed that CDK5 might be a critical player in cancer cell proliferation [12, 22]. CDK5 also showed to promote prostate cancer cell growth through androgen receptor [33]. Pozo et al. found that CDK5 was crucial for human medullary thyroid carcinoma cell proliferation and thus it contributed to the progression of medullary thyroid carcinoma [12]. Xu et al. also demonstrated that CDK5 and its two major binding partners, KIAA0528 and FIBP, were required for breast cancer cell growth and migration [22]. The important role of CDK5 in glioma cell growth was also shown in the studies described previously by Liu et al. and Catania et al. [15, 32]. Additionally, the strong correlation between CDK5 expression and Ki-67 LI was also found in our study, which disclosed that CDK5 could be involved in glioma cell proliferation. In support of the previous studies, our result suggests that CDK5 may regulate multiple cellular processes and contribute to tumorigenesis by promoting tumor proliferation and deterioration in glioma.

## Conclusions

Our results suggest that CDK5 may represent a valuable predictive marker of tumorigenesis and progression in glioma. Larger studies are still desirable in the future to provide stronger evidence for CDK5 as a candidate diagnostic and prognostic biomarker of glioma.

## Abbreviations

AKT: protein kinase B
ANOVA: analysis of variance
CDK5: cyclin-dependent kinase 5
Dab: the disabled-1
DLC1: deleted in liver cancer 1
EGFR: epidermal growth factor receptor
FAK: focal adhesion kinase
FIBP: acidic fibroblast growth factor intracellular-binding protein
GBM: glioblastoma multiform
GFAP: glial fibrillary acidic protein
IDH1: isocitrate dehydrogenase 1
LI: labeling index
MGMT: methyl-guanine-DNA methyltransferase
NSCLC: non-small- cell lung carcinoma
PIKE-A: isoform A of phosphatidylinositol 3-kinase enhancer
p-PPARγ: phospho peroxisome proliferator-activated receptors γ
RB: retinoblastoma protein
ROC curve: receiver operating characteristic curve
